# A Digital Intervention for Australian Adolescents Above a Healthy Weight (Health Online for Teens): Protocol for an Implementation and User Experience Study

**DOI:** 10.2196/13340

**Published:** 2019-10-10

**Authors:** Carly Jane Moores, Anthony Maeder, Jacqueline Miller, Ivanka Prichard, Lucy Kate Lewis, Lucinda Kate Bell, Aimee Macoustra, Michelle D Miller

**Affiliations:** 1 Nutrition and Dietetics College of Nursing and Health Sciences Flinders University Adelaide Australia; 2 Digital Health Research Centre College of Nursing and Health Sciences Flinders University Adelaide Australia; 3 Healthy Mothers Babies and Children South Australian Health and Medical Research Institute Adelaide Australia; 4 Health Sciences College of Nursing and Health Sciences Flinders University Adelaide Australia; 5 Sport, Health, Activity, Performance and Exercise Research Centre Flinders University Adelaide Australia; 6 Physiotherapy College of Nursing and Health Sciences Flinders University Adelaide Australia

**Keywords:** adolescent, overweight, diet, exercise behavior

## Abstract

**Background:**

More than one-fourth of Australian adolescents are overweight or obese, with obesity in adolescents strongly persisting into adulthood. Recent evidence suggests that the mid-teen years present a final window of opportunity to prevent irreversible damage to the cardiovascular system. As lifestyle behaviors may change with increased autonomy during adolescence, this life stage is an ideal time to intervene and promote healthy eating and physical activity behaviors, well-being, and self-esteem. As teenagers are prolific users and innate adopters of new technologies, app-based programs may be suitable for the promotion of healthy lifestyle behaviors and goal setting training.

**Objective:**

This study aims to explore the reach, engagement, user experience, and satisfaction of the new app-based and Web-based Health Online for Teens (HOT) program in a sample of Australian adolescents above a healthy weight (ie, overweight or obese) and their parents.

**Methods:**

HOT is a 14-week program for adolescents and their parents. The program is delivered online through the Moodle app–based and website-based learning environment and aims to promote adolescents’ lifestyle behavior change in line with Australian Dietary Guidelines and Australia’s Physical Activity and Sedentary Behaviour Guidelines for Young People (aged 13-17 years). HOT aims to build parental and peer support during the program to support adolescents with healthy lifestyle behavior change.

**Results:**

Data collection for this study is ongoing. To date, 35 adolescents and their parents have participated in one of 3 groups.

**Conclusions:**

HOT is a new online-only program for Australian adolescents and their parents that aims to reduce cardiovascular disease risk factors. This protocol paper describes the HOT program in detail, along with the methods to measure reach, outcomes, engagement, user experiences, and program satisfaction.

**Trial Registration:**

Australian New Zealand Clinical Trials Registry ACTRN12618000465257; https://www.anzctr.org.au/Trial/Registration/TrialReview.aspx?id=374771

**International Registered Report Identifier (IRRID):**

DERR1-10.2196/13340

## Introduction

### The Issue: Adolescent Obesity

At present, more than one-fourth of Australian adolescents aged 14 to 17 years are overweight or obese, which is significantly higher than 20 years ago [1]. High body mass index (BMI) in adolescence is difficult to reverse and persists into adulthood [2-5]. Previous findings from the American Bogalusa Heart Study, a longitudinal study with a mean follow-up of 17.6 years, showed the prevalence of obesity in adulthood was 86% for men and 90% for women among adolescents who had been obese between the ages of 15 and 17 years [4]. Adolescent obesity is associated with considerable short-term and long-term health consequences, such as increased risk of heart disease and diabetes [6,7]. These risk factors have also been shown to track into adulthood [2], which, in addition to the risk of being an obese adult, indicate a double burden of adolescent obesity on cardiovascular disease risk.

### Why Target Adolescence?

Adolescence is a period of transition during which autonomy and independence increase. During this life stage, autonomy over food choice [8] and influence from peers can contribute to overweight risk behaviors, including unhealthy diets, insufficient physical activity, and excessive sedentary time [9,10]. Parent behaviors, healthy home food environments [11], peer support from friends [12], and social norms [13] can each influence adolescent lifestyle behaviors and are important to consider in developing interventions for this population. Typical changes to diet during adolescence include a decrease in breakfast consumption and increased frequency of snacking, fast food consumption, and eating outside of the home environment [14-16]. As a result, diet quality declines from childhood to adolescence [17]. Activity changes include a decrease in physical activity (especially in girls) and an increase in sedentary time [14,18,19]. As lifestyle behaviors are pliable and behaviors formed during adolescence have been shown to track into adulthood [3,14], it is important to intervene during this time to promote healthier behaviors. It has been recently suggested that mid-teen years represent a tipping point as the window of opportunity to prevent irreversible damage to the cardiovascular system caused by unhealthy lifestyle factors and excess BMI may close after this time [20].

### Adolescents and Technology-Based Programs: The Evidence Gap

Adolescents are early adopters of technology and generally are innately accepting of innovative methods of communication and learning. In 2015, it was estimated that 65% of Australian teenagers aged 14 to 17 years used a mobile phone to access the internet, 74% used a computer to access the internet, and 80% had a smartphone [21]. Moreover, Australian adolescents aged between 15 and 17 years are the highest proportion of internet users (98%) [22]. Online programs have the capacity to achieve greater reach than face-to-face programs, as participants can be included irrespective of geography or means of transport to a physical location [23]. Although online-only programs exist for adult weight management [23], there is a paucity of online-only programs for secondary prevention of obesity in adolescents [24].

### The Health Online for Teens Program

A new program, Health Online for Teens (HOT), is the first Australian online-only, expert-supported group intervention involving parental and peer support for obesity prevention in adolescents. HOT is underpinned by theories of behavior change and self-determination and recognizes the importance of engagement in lifestyle choices at a critical, yet pliable, period of transition. Covering the key areas of overcoming peer pressure, maintaining a healthy diet, and being physically active as well as emotional well-being, HOT provides opportunities for teens (and their parents) to gain improved lifestyles through goal setting and peer and expert support.

### Study Aims

The objective of this study is to determine the feasibility of a new online healthy lifestyle program (HOT) to improve lifestyle-related behaviors in a sample of Australian adolescents above a healthy weight. Specifically, this study aims to (1) obtain feedback from a sample of overweight or obese teens who will be asked to discuss the content, features, and design of HOT and (2) determine the feasibility of HOT through pilot testing in a sample of up to 45 adolescents and collecting data on recruitment, retention, and engagement over the course of the 14-week program. This publication describes the study methods and rationale to achieve these research aims.

## Methods

### Design and Research Objectives

This study is a nonrandomized intervention feasibility trial [25] that aims to assess the acceptability, demand, implementation, and practicality of the HOT program. Accordingly, there is no control or comparator group in this study. The research protocol was prospectively registered with the Australian New Zealand Clinical Trials Registry (ACTRN12618000465257) on March 29, 2018. Ethics approval for this study was granted by the Flinders University Social and Behavioural Research Ethics Committee on March 21, 2018 (Project number 7896; Health Online for Teens: An Australian technology-based lifestyle program for overweight adolescents).

This paper reports the protocol of intervention and evaluation of HOT in line with the Standard Protocol Items: Recommendations for Interventional Trials (SPIRIT) checklist (Multimedia Appendix 1) [26,27]. The findings of the study will be reported according to the Transparent Reporting of Evaluations with Nonrandomized Designs Statement [28]. Textbox 1 outlines the research objectives of this feasibility study.

**Research objectives in the Health Online for Teens (HOT) feasibility study.**

- To assess engagement and use of the HOT program and its components and Health Online for Teens chat-bot (HOT-BOT) by teenage participants
- To assess engagement and use of HOT and parent resources by parents or caregivers of the participants
- To determine the reach of HOT recruitment and representativeness of the target population
- To determine the effectiveness of the program to support teenagers to achieve healthy lifestyle goals and improve weight, diet and activity behaviors, and self-perception (outcome evaluation)
- To determine program satisfaction and process evaluation data from participants
- To conduct focus groups and/or interviews to deeply explore the participants’ HOT experience, thoughts on the content, appearance and design of the program and its elements, and barriers or enablers to engagement

### Population

Australian adolescents will be recruited from the community through social media advertising. Adolescent participants express interest to participate in the study through an online survey administered through Qualtrics (Qualtrics). Qualtrics is an online survey tool that is supported by the researchers’ host institution. Adolescents expressing an interest to participate will be screened against several inclusion and exclusion criteria. Eligible adolescents include girls and boys, aged between 13 and 17 years at enrollment, who are above a healthy weight for their age and gender, not pregnant or breastfeeding, with access to Wi-Fi at home. Eligible parents or caregivers of included adolescents can be of any age, and typically, 1 to 2 parents or caregivers are anticipated to be included for each adolescent. Adolescent participants will be identified as above a healthy weight (overweight, obese, and morbidly obese) using self-reported height and weight and weight status from BMI (International Obesity Task Force [IOTF] extended criteria) [29]. Groups are planned to commence when there are a minimum of 10 eligible adolescent and parent dyads who provide their consent to participate. Parents will provide informed consent for themselves and their child to participate, and children will provide informed assent to participate in the form of scanned hardcopy consent forms, electronically signed sheets, or upon commencement of baseline surveys administered online. Copies of participant information sheets and consent and assent forms are provided in Multimedia Appendix 2 as per the SPIRIT Checklist [26,27].

### Intervention

The online HOT program aims to support overweight or obese adolescents to improve their lifestyle, through setting goals and making sustainable changes to diet and activity patterns. The HOT program aims to improve knowledge on healthy diet, activity, and emotional well-being and build skills and capacity for teens to plan ahead, set their own goals, and reflect and evaluate on their progress. The HOT program encourages participants to seek support from parents and peers to facilitate a home improvement, which is supportive of a healthy lifestyle and self-esteem.

The HOT program has a number of principles that inform program targets and strategies that are promoted to achieve the targets (Table 1). These targets are underpinned by national guidelines [30-32] and other evidence-based recommendations [33-36]. The HOT program also aims to build capacity and resilience in teenagers to identify barriers toward a healthy lifestyle, plan ahead, and take small steps and sustainable changes to overcome these. The HOT program incorporates a number of behavior change techniques [37] including providing information on consequences of behavior in general, providing normative information about others’ behavior, goal setting (behavior); barrier identification or problem solving, prompting self-monitoring of behavior, prompting a focus on past success, providing information on where and when to perform the behavior, providing instruction on how to perform the behavior, using of follow-up prompts, planning social support or social change, and relapse prevention or coping planning.

Goal setting is a key element of HOT, and although HOT participants set their own goals around diet and activity and monitor their progress, HOT aims to address common adverse behaviors for adolescents, including skipping breakfast, frequent fast food intake, high levels of sedentary behavior, and low levels of physical activity, by encouraging individual goal setting in these areas. HOT employs the use of SMART goals [39] that are specific, measurable, achievable, realistic, and time bound. An outline of each week of the program, including the context for individuals to conceptualize their own SMART goals, is presented in Table 2. In addition to HOT sessions that are accessed through Moodle, a supportive and motivational chatbot (HOT-BOT) is built into the HOT program to collect information on teen goals and prompt them to complete the program tasks for the week, set their goals, and reflect on their progress. HOT-BOT is delivered through Facebook Messenger and operates on the Chatfuel platform [40]. Facebook Messenger was selected for the HOT-BOT, as Facebook is widely used and accepted by both adolescents and their parents. The Facebook Messenger platform is also compatible with the chatbot technology, whereas other social media platforms commonly used by adolescents do not support this technology (eg, SnapChat and Instagram).

**Table 1:** Health Online for Teens (HOT) lifestyle behavior principles, targets, and strategies for adolescents.

Domain	HOT principles	HOT targets	HOT strategies	References
Diet	Eat a wide range of core foods every day based on the Australian Guide to Healthy Eating (AGHE)	Aim to eat the following: 2 serves of fruits per day; 5 serves of vegetables per day; Wholegrain and wholemeal cereal-based products (bread, pasta, rice, and cereals); Low-fat dairy and lean meat products	Plan healthy meals and snacks ahead of time; Take a packed lunch to school; Include fruits and/or vegetables in every meal; Get involved in food planning, shopping, preparation, and cooking; Try healthy recipes; Encourage family meals	Australian Dietary Guidelines (ADG) [31] and the AGHE [32]; Larson *et al*, 2007 [38]
Diet	Limit discretionary foods and choose healthy snacks instead	Aim to limit the number of discretionary foods each week	Choose fruits and vegetables as snacks and swap discretionary foods (chips, chocolates, muesli, and snack bars) for healthy snacks; Plan snacks ahead of time and pack healthy snacks for school and other daily activities	ADG [31] and the AGHE [32]
Diet	Replace sweetened soft drinks, sports drinks, energy drinks, flavored milk, and cordial with water	Aim to drink 2 L of water per day and avoid sweetened beverages (cordial and soft drinks)	Pack a water bottle with you wherever you go; Refill at water fountains at school and when on the go; On hot days, freeze water overnight for a refreshing drink during the day	ADG [31] and the AGHE [32]; Healthy Kids (NSW Health) [33]
Physical activity	Be active every day	Aim to do at least 1 hour of moderate to vigorous intensity physical activity every day	Incorporate physical activity in everyday life; it is fun and a great way to spend time with people.	Australia’s Physical Activity and Sedentary Behaviour Guidelines [30]
Physical activity	Be active every day	Aim to include strengthening exercises in physical activity at least 3 times per week	Include activities that build strength for strong muscles and bones; these do not need to be gym-based.	Australia’s Physical Activity and Sedentary Behaviour Guidelines [30]
Sedentary behavior	Minimize time spent looking at screens (eg, TV, phone, computer, and iPad)	Aim for no more than 2 hours in screen-based activities (outside school hours and not including homework)	Plan specific periods of time for watching TV and using other screen devices; Plan for active and outdoor activities with friends over watching TV and playing computer games; Choose active travel options where possible	Australia’s Physical Activity and Sedentary Behaviour Guidelines [30]
Sedentary behavior	Minimize sedentary time	Limit prolonged periods of sitting (>30 min)	Try to get up and move regularly when at home and when possible at school	Australia’s Physical Activity and Sedentary Behaviour Guidelines [30]
Sleep	Get plenty of sleep each night	Aim for 8-10 hours of quality sleep per night	Avoid using screens in the bedroom and avoid screen use just before bedtime; Establish a relaxing bedtime routine	National Sleep Foundation (US) [34]; Australian Centre for Education in Sleep [36]; Better Health Channel (Vic Health) [35]
Well-being	Develop and maintain positive relationships with self, family, friends, and peers	Treat others with respect in the way you would like to be treated; Listen to and respect others’ points of view; Suggest solutions to problems and be encouraging; Avoid toxic relationships—online and in real life; Increase self-awareness and learn principles of mindfulness	Treat others with respect in the way you would like to be treated; Listen to and respect others’ points of view; Suggest solutions to problems and be encouraging; Avoid toxic relationships—online and in real life; Increase self-awareness and learn principles of mindfulness	—^a^
Well-being	Be a positive influence and encourage healthy behaviors in others	Encourage and support healthy behaviors; Be a role model for others; Share enthusiasm and positivity for healthy lifestyles	Encourage and support healthy behaviors; Be a role model for others; Share enthusiasm and positivity for healthy lifestyles	—

^a^Not applicable.

**Table 2:** Health Online for Teens (HOT) program outline.

Time			Weekly session content	Weekly activities
Week	Days in	Days to go		
Preprogram	−7 to 0	99 to 105	N/A^a^ evaluation and reflection	Preprogram evaluation
1	1 to 7	93 to 98	Introduction and welcome—live session: What is HOT?	Rules of communicating in HOT—dos and don’ts of participating in forums and chats; Introduction to your HOT group (forum)
1	1 to 7	93 to 98	How to find your way around HOT;	To do: introduction to your HOT group (forum)
1	1 to 7	93 to 98	Introduction to HOT Targets (Table 1)	Health Online for Teens chatbot (HOT-BOT) setup
2	8 to 14	85 to 91	Nutrition: how your diet impacts health; Introduction to the Australian Guide to Healthy Eating and the Australian Dietary Guidelines	To do: Healthy Eating Quiz [41] and forum discussion of results
2	8 to 14	85 to 91	Activity: what is physical activity and sedentary behavior?	To do: keep a physical activity and screen time diary
2	8 to 14	85 to 91	Well-being: benefits of peer and parental support	HOT-BOT check in
2	8 to 14	85 to 91	Making changes: setting SMART (Specific, Measurable, Achievable, Realistic, and Timely) goals and changing behaviors	
3	15 to 21	78 to 84	Nutrition: the 5 core food groups and the other discretionary foods and complete the food group quiz	To do: keep a diary of what foods you normally eat on a weekend day and weekday
3	15 to 21	78 to 84	Activity: what are the physical activities and sedentary behavior guidelines	To do: reflect on how much activity you do each day; compare weekday with weekend day
3	15 to 21	78 to 84	Well-being: finding and giving support and supportive environments	To do: identify barriers to healthy eating and possible solutions
3	15 to 21	78 to 84	Making changes: what are barriers and what are enablers?	My goals: set a SMART goal to be more physically active
3	15 to 21	78 to 84		HOT-BOT check in
4	22 to 28	71 to 77	Nutrition: healthy dietary fats	To do: reflect on your diet on weekend/nonweekend days: how does it compare?
4	22 to 28	71 to 77	Activity: benefits of physical activity	My goals: set a SMART goal to try a new physical activity with a friend or family member
4	22 to 28	71 to 77	Well-being: what is body image	HOT-BOT check in
4	22 to 28	71 to 77	Making changes: reviewing your goals: are they SMART?	
5	29 to 35	64 to 70	Nutrition: importance of breakfast; healthy breakfast ideas	To do: body functionality writing task (modified from [42])
5	29 to 35	64 to 70	Activity: barriers to physical activity (busy lives, neighborhoods, and safety) and tips for exercising safely in the neighborhood	My goals: set a SMART goal to: eat a healthy breakfast and try a physically active alternative to watching TV or playing videogames with a friend
5	29 to 35	64 to 70	Well-being: positive body image	HOT-BOT check in
5	29 to 35	64 to 70	Making changes: being realistic	
6	36 to 42	57 to 63	Nutrition: healthy lunches and snacks for school	My goals: set a SMART goal to: improve lunches and snacks for school this week and try a new strengthening exercise
6	36 to 42	57 to 63	Activity: strength building activities and what makes us strong	HOT-BOT check in
6	36 to 42	57 to 63	Well-being: physical activity and health and mental well-being	
6	36 to 42	57 to 63	Making changes: goal setting review	
7	43 to 49	50 to 56	Nutrition: proportion of food groups in balanced dinner meals and healthy recipe videos	My goals: set a SMART goal to: cook dinner for your family one night this week and reduce screen time this week
7	43 to 49	50 to 56	Activity: strategies to reduce (nonhomework) screen time	HOT-BOT check in
7	43 to 49	50 to 56	Well-being: building resilience	
7	43 to 49	50 to 56	Making changes: building on your goals	
8	50 to 56	43 to 49	Nutrition: choosing healthier takeaway foods	To do: watch the Dove real beauty campaign and think about how the media can control what we see
8	50 to 56	43 to 49	Activity: do you spend too much time attached to devices (eg, checking social media and texting)?	To do: find and appraise a source of nutrition or physical activity advice on social media
8	50 to 56	43 to 49	Well-being: how can media/social media makes us feel and why?	My goals: set a SMART goal to: choose a healthier takeaway meal and use social media less this week
8	50 to 56	43 to 49	Making changes: review at the halfway point	HOT-BOT check in
9	57 to 63	36 to 42	Nutrition: choose water over sugary drinks	To do: sugar drinks quiz
9	57 to 63	36 to 42	Activity: choose active transport over inactive options	To do: keep a sleep diary this week
9	57 to 63	36 to 42	Well-being: being cyberaware and being safe online	My goals: set a SMART goal to: drink more water/less soft drink and take an active transport option
9	57 to 63	36 to 42	Making changes: how to stay motivated	HOT-BOT check in
10	64 to 70	29 to 35	Nutrition: label reading and selecting items	To do: read labels of some common foods/snacks/drinks at home
10	64 to 70	29 to 35	Activity: what are the sleep recommendations	My goals: set a SMART goal to improve your sleep
10	64 to 70	29 to 35	Well-being: overcoming peer pressure	HOT-BOT check in
10	64 to 70	29 to 35	Making changes: how to cope with relapse	
11	71 to 77	22 to 28	Nutrition: healthy and easy food swaps at home and away and recipe modification	To do: complete the sleeping habits checklist quiz
11	71 to 77	22 to 28	Activity: sleeping habits and sleep hygiene	My goals: set a SMART goal to choose a recipe and make it healthier
11	71 to 77	22 to 28	Well-being: increasing your self-awareness	HOT-BOT check in
11	71 to 77	22 to 28	Making changes: how to prevent relapse, healthy eating at parties and celebrations	
12	78 to 84	15 to 21	Nutrition: why we eat—hunger and satiety, mindful eating	To do: reflect on why you eat (mindless eating reflection task)
12	78 to 84	15 to 21	Well-being: self-awareness and mindfulness	To do: eat a meal mindfully and intuitively
12	78 to 84	15 to 21	Making changes: sustainability and long-term changes	My goals: set a SMART goal to make changes to your sleep routine
12	78 to 84	15 to 21		HOT-BOT check in
13	85 to 91	8 to 14	Nutrition/activity/well-being: Review of HOT targets; Lifestyle behaviors can be related to each other; Bringing all these behaviors together to have and maintain a healthy lifestyle	To do: mindful physical activity exercise
13	85 to 91	8 to 14	Making changes: maintaining healthy lifestyle changes and relationships	My goals: set SMART lifestyle (diet and activity) goals you want to maintain after HOT
13	85 to 91	8 to 14		To do: think about how you can maintain goals and HOT target behaviors (identify barriers and enablers)
13	85 to 91	8 to 14		HOT-BOT check in
14	93 to 98	1 to 7	Program review—what have we all achieved?—live session	To do: Healthy Eating Quiz and reflection on diet changes during HOT
14	93 to 98	1 to 7	How far have we come and where to from here	To do: review of goals for after HOT
14	93 to 98	1 to 7	Where to get help/support after HOT	HOT-BOT goodbye
Postprogram	99 to 105	−7 to 0	N/A evaluation and reflection	Postprogram evaluation

^a^N/A: not available.

### Program Access

Adolescents participating in HOT will be loaned an iPad Mini for the duration of the project. The iPad is configured with the apps required for the project (Moodle and Facebook Messenger) and will be delivered to the adolescents approximately 1.5 weeks before they start the program. On safe return of equipment at the end of the program, families will receive an Aus $50 gift card for either Apple iTunes/App Store or Coles Myer in recognition of their contribution to this study. Adolescents will be provided with their HOT login and will be able to log in to Moodle from any other device as they wish. Parents will not be loaned any device during HOT but will be given a login to access HOT for themselves using their own personal devices (smartphone or tablet) or computer (desktop or laptop). Parents and adolescents will have access to the same HOT content; however, there will be separate spaces for parents and adolescents to connect with their peers through a discussion forum. In addition to HOT sessions, parents will have access to specifically tailored information including supporting your teen to meet HOT targets, communicating with your teen, your teen and body image, healthy lifestyle guidelines for adults (diet, activity, and sleep), and a collection of additional parent resources (Web links).

### Evaluation

A mixed-methods approach comprising quantitative and qualitative evaluation will be used to evaluate the feasibility of HOT. This project aims to evaluate the feasibility of HOT, including population characteristics (program reach), program outcomes (indication of program effectiveness), processes (program implementation), engagement (program use), and acceptability (program satisfaction) measures. A summary of measures and the time of assessment are described in Multimedia Appendix 3. Specific primary outcomes and secondary outcomes of interest are listed in Table 3.

**Table 3:** Primary and secondary outcomes and their assessment.

Outcomes		Assessment method	Time point
**Primary outcomes**			
	User engagement (adolescents and parents) with the program	Access of 14 program sessions, expressed as a proportion of content covered as measured by Moodle metrics	From the start to the end of the program (14 weeks)
	Adolescent self-reported weight status	Height and weight used to calculate BMIz^a^ [43] and International Obesity Task Force extended weight status [29]	Change from preprogram (enrollment) to postprogram (after 14-week intervention)
**Secondary outcomes**			
	Adolescent diet behavior	Children's Dietary Questionnaire [44] and Serves of Core Foods [45]	Change from preprogram (enrolment) to postprogram (after 14-week intervention)
	Adolescent physical activity behavior	7-day 24-hour accelerometry, GENEActiv Original wrist-worn accelerometer [46] on nondominant hand [47] and Adolescent Physical Activity Recall Questionnaire [48]	Change from preprogram to postprogram (after 14-week intervention)
	Adolescent sedentary behavior	7-day 24-hour accelerometry, GENEActiv wrist-worn accelerometer [46] on nondominant hand [47] and Adolescent Sedentary Activity Questionnaire [49]	Change from preprogram (enrollment) to postprogram (immediately after a 14-week intervention)
	Adolescent self-perception	Harter self-perception profile for adolescents [50]	Change from preprogram (enrollment) to postprogram (immediately after a 14-week intervention)
	Parent and adolescent program satisfaction	Purpose-designed questionnaire	Postprogram (immediately after the 14-week intervention)
	Parent and adolescent program satisfaction	Qualitative interviews	Postprogram

^a^BMIz: body mass index z-score.

#### Quantitative Evaluation: Outcomes

Program outcomes will be determined through pre-post program changes in lifestyle behaviors associated with increased cardiovascular risk and obesity (weight, diet, physical activity, and sedentary screen time) as well as changes in adolescent self-perception domains and overall self-esteem. Adolescents will be asked to complete online preprogram and postprogram semiquantitative surveys that assess key lifestyle behaviors. Self-reported adolescent height and weight will be used to calculate BMI z-score [43] and IOTF extended weight status categories [29]. Child diet will be measured by the Children's Dietary Questionnaire [44] and estimation of Serves of Core Foods [45], which will be compared with Australian Dietary Guidelines [31] and recommended serves of core and discretionary foods as per the Australian Guide to Healthy Eating [32]. Objective measurement of physical activity and sedentary time will be collected by 7-day 24-hour accelerometry, GENEActiv Original wrist-worn accelerometer [46] on nondominant hand [47]. Total time spent in sedentary, light, moderate, and physical activity will be explored. Additional activity data will be collected using the Adolescent Physical Activity Recall Questionnaire [48] and Adolescent Sedentary Activity Questionnaire [49]. Activity behaviors before and after the program will be compared with Australia’s Physical Activity and Sedentary Behaviour Guidelines for Young People (aged 13-17 years) [30]. Self-perception and self-esteem will be collected using the Harter self-perception profile for adolescents [50]. This tool explores 8 specific self-concept domains: scholastic competence, athletic competence, social competence, physical appearance, behavioral conduct, close friendship, romantic appeal, and job competence, as well as a ninth subscale that reports global self-worth [51].

#### Quantitative Evaluation: Engagement and Program Satisfaction

Usage of the program will be measured using metrics, together with program satisfaction evaluation will inform adolescent engagement with the program elements and its acceptability. Data on program engagement for both parents and teens will be obtained from the online metrics collected by Moodle and Chatfuel, where applicable. Usage metrics will include weekly sessions viewed (n, %), total number of hits (n), average number of hits per week (n, %), number of individual sessions on Moodle (n), number of forum posts (n), and number of chatbot weekly check-ins completed (n, %).

Parent and adolescent satisfaction with the HOT program will be explored by a purpose-designed questionnaire that is distributed postprogram.

#### Quantitative Evaluation: Data Management and Analysis

Participants will be allocated unique identification (ID) numbers at enrollment, and all data collected will be recorded against this ID number; hence, evaluation data will be deidentified for research purposes. All questionnaires will be completed online through Qualtrics. The collection of evaluation data online has considerable advantages by avoiding manual data entry by research staff, minimizing data entry errors and staff time. Direct data entry also enables the research team immediate access to the data by downloading directly to IBM SPSS v23 (IBM Corp). As there is no control group for this healthy lifestyle intervention, preprogram and postprogram changes in lifestyle measures and child anthropometric data will be reported descriptively. Continuous data will be analyzed in SPSS and reported as means (95% CI lower limit to upper limit) or medians (interquartile range), as appropriate, and categorical data will be reported as n (%). Repeated-measures paired *t* tests will be used to analyze changes in outcomes over time for parametric data, whereas the Wilcoxon signed-rank test will be used for paired pre-post nonparametric data. The alpha value will be set at .05. The proportion of adolescents meeting recommendations for diet, physical activity, and sedentary time as per Australian Guidelines [30] before and after the program will also be described. Finally, associations between program engagement and participants’ characteristics and program outcomes will be explored.

#### Qualitative Analysis: Data Collection and Analysis

On completion of HOT, parents and adolescents may be invited to participate in an interview or focus group to more deeply explore user experience in HOT. Qualitative semistructured interviews and/or focus groups will be conducted face-to-face for those living in the Adelaide area or over the phone for rural, remote, and interstate participants. Interview schedules will broadly aim to explore the user experience and usefulness of HOT for both adolescents and their parents. Interview questions will probe experiences for adolescents and their parents and will aim to capture what participants were seeking when they enrolled into HOT (including perception of lifestyle problems and their severity), what changes they have made to their lifestyle as a result of participating in HOT, what challenges to making behavior changes were encountered when participating in HOT, and what additional content and/or support would have helped to get more out of the HOT program, including to make suggested lifestyle behavior changes. A more detailed indicative interview schedule is presented in Table 4. It is anticipated that these participant interviews will elucidate suggestions for improvement of HOT, including HOT content and program delivery to optimize user’s experience and satisfaction.

Qualitative semistructured interviews and/or focus groups will be audio-recorded and transcribed verbatim. Data will be entered and coded in NVivo version 10 qualitative data analysis software (QSR International Pty Ltd) and analyzed thematically. All data, including qualitative data, will be deidentified and stored in a secure, password-protected drive with access only available to the research team members.

**Table 4:** Indicative questions for parents and adolescents following participation in Health Online for Teens (HOT).

Domain	Questions for parents	Questions for adolescents
Introduction	Can you tell me a bit about yourself and your family?; How would you describe your relationship with [*adolescent name*]?; How would you describe your family lifestyle? (prompts for lifestyle behaviors: diet, physical activity, sedentary behavior, sleep, wellbeing)	Can you tell me a bit about yourself and your family?; How would you describe your relationship with your parent/s? Siblings?; How would you describe your family lifestyle? (prompts for lifestyle behaviors: diet, physical activity, sedentary behavior, sleep, wellbeing)
Motivations for enrollment	Can you tell me a little bit about how you became involved in HOT? (prompt: whose idea was it to be involved; how did you feel); Can you tell me about what you expected HOT to be like?; What was [*adolescent name*] hoping to get from HOT?; Can you tell me about [*adolescent name*]’s health?	Can you tell me a little bit about how you became involved in HOT?; Can you tell me about what you expected HOT to be like?; What were you hoping to get from HOT?; Can you tell me about your health?
Lifestyle changes	How motivated do you think [*adolescent name*] was to make changes during HOT?; How would you describe [*adolescent name*]’s lifestyle before HOT? (prompts for lifestyle behaviors: diet, physical activity, sedentary behavior, sleep, wellbeing); Do you think that there have been changes to [*adolescent name*]’s lifestyle during HOT? (prompts for lifestyle behaviors: diet, physical activity, sedentary behavior, sleep, wellbeing); Can you tell me how HOT has impacted your family lifestyle? (prompts for lifestyle behaviors: diet, physical activity, sedentary behavior, sleep, wellbeing); Follow-up probing questions related to making changes: Were there things which helped you to make these changes? Were there any things which made it harder for you to make the changes you wanted?	Can you tell me about your motivation to make changes during HOT? How would you describe your lifestyle before HOT? (prompts for lifestyle behaviors: diet, physical activity, sedentary behavior, sleep, wellbeing); Can you tell me about your lifestyle now? (prompts for lifestyle behaviors: diet, physical activity, sedentary behavior, sleep, wellbeing); Follow-up probing questions related to making changes: Were there things which helped you to make these changes? Were there any things which made it harder for you to make the changes you wanted?
Influences	Can you tell me who you think [*adolescent name*] looks to for healthy lifestyle advice?	Can you tell me where you go to get healthy lifestyle advice? (prompt: role models)
Parent role	What do you think your role was in HOT?; What are some examples of how you helped support [*adolescent name*] during HOT?; Can you describe to me what you think a parent’s role is in their teenagers’ health?	Can you tell me about the role of your parent in HOT?; Can you describe to me what you think a parent’s role is in their teenagers’ health?
Experiences with HOT	How would you describe your experience with HOT?; What are some of the things you liked about HOT? (prompt: and what about for [*adolescent name*]); What are some of the things you didn’t like about HOT? (prompt: and what about for [*adolescent name*])	How would you describe your experience with HOT?; What are some of the things you liked about HOT?; What are some of the things you didn’t like about HOT?
Time spent on HOT	Can you tell me a bit about how and when you used HOT?; Are there any suggestions for changes to HOT which would have improved the program for you? (prompt: and what about for [*adolescent name*]); Are there additional supports which would have helped you in HOT? (prompt: and what about for [*adolescent name*])	Can you tell me a bit about how and when you used HOT?; Are there any suggestions for changes to HOT which would have improved the program for you?; Are there additional supports which would have helped you in HOT? (prompt: and what about for [*adolescent name*])

### Project Governance

The authors of this paper comprise the multidisciplinary steering committee for the project that includes an advanced accredited practicing dietitian, accredited practicing dietitians, a registered nutritionist, a physiotherapist, and health psychology and digital health experts. The project governance will also comprise a 4-member Expert Advisory Committee comprising the HOT project manager including 3 Australian experts in the areas of nutrition and dietetics, physical activity, and electronic health and behavior change and are external to the administering institution. Any publications arising from this study will be reviewed by all members of the steering committee before submission, and authorship will be decided according to contribution.

## Results

Data collection for this study is ongoing. To date, 35 adolescents and their parents have participated in one of 3 groups.

## Discussion

Multiple health risk behaviors are recognized to emerge and cluster during the adolescent life stage, including cigarette smoking, alcohol consumption, and drug use [52]. Although there are laws and public health strategies to address these behaviors in Australia, there is a lack of community programs and support for overweight or obese teenagers to make well-informed lifestyle decisions. Unlike younger children whom have had greater improvements in health over the past 50 years, teens are a comparatively underserved community group [53]. This project is a pilot feasibility study of a new, innovative, evidence-based approach to address the public health priority of adolescent obesity, which aligns with recommendations from the World Health Organization Commission on Ending Childhood Obesity to provide family-based, multicomponent, lifestyle weight management services for children and young people who are obese as part of universal child health care [54]. It is crucial to intervene and increase healthy lifestyle behaviors during the teenage years to prevent irreversible cardiovascular damage [20] and minimize heart health risks.

Adolescent obesity remains a public health concern in Australia and many other Western nations. This study aims to explore the feasibility of the new online program HOT that promotes lifestyle behavior change for adolescents who are above a healthy weight. If this program is deemed to be feasible and acceptable for adolescents and their parents, program effectiveness will be explored in a subsequent randomized controlled trial. It is important to note that while preliminary pre-post outcome measures will be collected in this sample, these data will be used to inform sample size calculations for a larger randomized controlled trial rather than implying intervention effectiveness. Feedback from users will be incorporated in future intervention delivery where possible to improve the experience of future users.

## Appendix

Multimedia Appendix 1 Standard Protocol Items: Recommendations for Interventional Trials (SPIRIT) checklist and items.

Multimedia Appendix 2 Information sheet for teens and parents, consent form for teens and parents, assent form for teens.

Multimedia Appendix 3 Schedule of enrolment, interventions, and assessments (SPIRIT template).

## Abbreviations

BMI: body mass index
HOT: Health Online for Teens
ID: identification
IOTF: International Obesity Task Force
SPIRIT: Standard Protocol Items: Recommendations for Interventional Trials

## References

[1] (2017). Australian Institute of Health and Welfare.

[2] Celermajer DS, Ayer JG (2006). Childhood risk factors for adult cardiovascular disease and primary prevention in childhood. Heart.

[3] Craigie AM, Lake AA, Kelly SA, Adamson AJ, Mathers JC (2011). Tracking of obesity-related behaviours from childhood to adulthood: a systematic review. Maturitas.

[4] Freedman DS, Khan LK, Serdula MK, Dietz WH, Srinivasan SR, Berenson GS (2005). The relation of childhood BMI to adult adiposity: the Bogalusa Heart Study. Pediatrics.

[5] Singh AS, Mulder C, Twisk JW, van Mechelen W, Chinapaw MJ (2008). Tracking of childhood overweight into adulthood: a systematic review of the literature. Obes Rev.

[6] Kelsey MM, Zaepfel A, Bjornstad P, Nadeau KJ (2014). Age-related consequences of childhood obesity. Gerontology.

[7] Pulgarón ER (2013). Childhood obesity: a review of increased risk for physical and psychological comorbidities. Clin Ther.

[8] Bassett R, Chapman GE, Beagan BL (2008). Autonomy and control: the co-construction of adolescent food choice. Appetite.

[9] Badaly D (2013). Peer similarity and influence for weight-related outcomes in adolescence: a meta-analytic review. Clin Psychol Rev.

[10] Fitzgerald A, Fitzgerald N, Aherne C (2012). Do peers matter? A review of peer and/or friends' influence on physical activity among American adolescents. J Adolesc.

[11] Arcan C, Neumark-Sztainer D, Hannan P, van den Berg P, Story M, Larson N (2007). Parental eating behaviours, home food environment and adolescent intakes of fruits, vegetables and dairy foods: longitudinal findings from project EAT. Public Health Nutr.

[12] Rosenrauch S, Ball K, Lamb KE (2017). Associations between perceived friends' support of healthy eating and meal skipping in adolescence. Public Health Nutr.

[13] Draper CE, Grobler L, Micklesfield LK, Norris SA (2015). Impact of social norms and social support on diet, physical activity and sedentary behaviour of adolescents: a scoping review. Child Care Health Dev.

[14] Araújo J, Ramos E (2017). Paediatric obesity and cardiovascular risk factors – a life course approach. Porto Biomed J.

[15] Alexy U, Wicher M, Kersting M (2010). Breakfast trends in children and adolescents: frequency and quality. Public Health Nutr.

[16] Feeley A, Musenge E, Pettifor JM, Norris SA (2012). Changes in dietary habits and eating practices in adolescents living in urban South Africa: the birth to twenty cohort. Nutrition.

[17] Story M, Neumark-Sztainer D, French S (2002). Individual and environmental influences on adolescent eating behaviors. J Am Diet Assoc.

[18] Collings PJ, Wijndaele K, Corder K, Westgate K, Ridgway CL, Sharp SJ, Atkin AJ, Bamber D, Goodyer I, Brage S, Ekelund U (2015). Prospective associations between sedentary time, sleep duration and adiposity in adolescents. Sleep Med.

[19] Dumith SC, Gigante DP, Domingues MR, Kohl 3rd HW (2011). Physical activity change during adolescence: a systematic review and a pooled analysis. Int J Epidemiol.

[20] Hanvey AN, Mensah FK, Clifford SA, Wake M (2017). Adolescent cardiovascular functional and structural outcomes of growth trajectories from infancy: prospective community-based study. Child Obes.

[21] (2016). Australian Communications Media Authority.

[22] Adair LS, Gordon-Larsen P, Du SF, Zhang B, Popkin BM (2014). The emergence of cardiometabolic disease risk in Chinese children and adults: consequences of changes in diet, physical activity and obesity. Obes Rev.

[23] Kozak AT, Buscemi J, Hawkins MA, Wang ML, Breland JY, Ross KM, Kommu A (2017). Technology-based interventions for weight management: current randomized controlled trial evidence and future directions. J Behav Med.

[24] Moores CJ, Bell LK, Miller J, Damarell RA, Matwiejczyk L, Miller MD (2018). A systematic review of community-based interventions for the treatment of adolescents with overweight and obesity. Obes Rev.

[25] Bowen DJ, Kreuter M, Spring B, Cofta-Woerpel L, Linnan L, Weiner D, Bakken S, Kaplan CP, Squiers L, Fabrizio C, Fernandez M (2009). How we design feasibility studies. Am J Prev Med.

[26] Chan AW, Tetzlaff JM, Altman DG, Laupacis A, Gøtzsche PC, Krleža-Jerić K, Hróbjartsson A, Mann H, Dickersin K, Berlin JA, Doré CJ, Parulekar WR, Summerskill WS, Groves T, Schulz KF, Sox HC, Rockhold FW, Rennie D, Moher D (2013). SPIRIT 2013 statement: defining standard protocol items for clinical trials. Ann Intern Med.

[27] Chan AW, Tetzlaff JM, Gøtzsche PC, Altman DG, Mann H, Berlin JA, Dickersin K, Hróbjartsson A, Schulz KF, Parulekar WR, Krleza-Jeric K, Laupacis A, Moher D (2013). SPIRIT 2013 explanation and elaboration: guidance for protocols of clinical trials. Br Med J.

[28] des Jarlais DC, Lyles C, Crepaz N, TREND Group (2004). Improving the reporting quality of nonrandomized evaluations of behavioral and public health interventions: the TREND statement. Am J Public Health.

[29] Cole TJ, Lobstein T (2012). Extended international (IOTF) body mass index cut-offs for thinness, overweight and obesity. Pediatr Obes.

[30] (2014). Australian Government Department of Health.

[31] (2013). Eat For Health.

[32] (2013). Eat For Health.

[33] (2018). Healthy Kids: NSW Government.

[34] (2018). National Sleep Foundation.

[35] (2014). Better Health Channel.

[36] (2008). Australian Center for Education in Sleep.

[37] Michie S, Ashford S, Sniehotta FF, Dombrowski SU, Bishop A, French DP (2011). A refined taxonomy of behaviour change techniques to help people change their physical activity and healthy eating behaviours: the CALO-RE taxonomy. Psychol Health.

[38] Larson NI, Neumark-Sztainer D, Hannan PJ, Story M (2007). Family meals during adolescence are associated with higher diet quality and healthful meal patterns during young adulthood. J Am Diet Assoc.

[39] Doran GT (1981). Temple MIS.

[40] (2018). Chatfuel.

[41] (2013). Healthy Eating Quiz.

[42] Alleva JM, Martijn C, van Breukelen GJ, Jansen A, Karos K (2015). Expand Your Horizon: a programme that improves body image and reduces self-objectification by training women to focus on body functionality. Body Image.

[43] Kuczmarski RJ, Ogden CL, Guo SS, Grummer-Strawn LM, Flegal KM, Mei Z, Wei R, Curtin LR, Roche AF, Johnson CL (2002). 2000 CDC growth charts for the United States: methods and development. Vital Health Stat 11.

[44] Magarey A, Golley R, Spurrier N, Goodwin E, Ong F (2009). Reliability and validity of the children's dietary questionnaire; a new tool to measure children's dietary patterns. Int J Pediatr Obes.

[45] Golley RK, Magarey AM, Daniels LA (2011). Children's food and activity patterns following a six-month child weight management program. Int J Pediatr Obes.

[46] (2017). Activinsights.

[47] Esliger DW, Rowlands AV, Hurst TL, Catt M, Murray P, Eston RG (2011). Validation of the GENEA Accelerometer. Med Sci Sports Exerc.

[48] Booth ML, Okely AD, Chey TN, Bauman A (2002). The reliability and validity of the adolescent physical activity recall questionnaire. Med Sci Sports Exerc.

[49] Hardy LL, Booth ML, Okely AD (2007). The reliability of the adolescent sedentary activity questionnaire (ASAQ). Prev Med.

[50] Harter S (2012). DU Portfolio - University of Denver.

[51] Harter S (2012). DU Portfolio - University of Denver.

[52] Kipping RR, Campbell RM, MacArthur GJ, Gunnell DJ, Hickman M (2012). Multiple risk behaviour in adolescence. J Public Health (Oxf).

[53] Sawyer SM, Afifi RA, Bearinger LH, Blakemore SJ, Dick B, Ezeh AC, Patton GC (2012). Adolescence: a foundation for future health. Lancet.

[54] (2016). World Health Organization.

