# Supplementary material for: A Digital Intervention for Australian Adolescents Above a Healthy Weight (Health Online for Teens): Protocol for an Implementation and User Experience Study
Source: JMIR Res Protoc. 2019 Oct 10;8(10):e13340. doi: 10.2196/13340 (PMC6913688; doi:10.2196/13340)
Supplement: Multimedia Appendix 2 [file resprot_v8i10e13340_app2.pdf]

Multimedia Appendix 3: Information sheet for teens and parents, consent form for teens and parents, assent form for teens

College of Nursing
& Health Sciences

Health Online for Teens
(HOT)

Nutrition and Dietetics
College of Nursing and
Health Sciences
Flinders University
GPO Box 2100
Adelaide SA 5001

HOT@flinders.edu.au

INFORMATION SHEET
for adolescents

Title: Testing a new online program to improve teenage eating and lifestyle behaviours

Researchers:

Professor Michelle Miller
College of Nursing and Health Sciences
Flinders University
Ph: +61 8 8201 2421

Professor Anthony Maeder
Health Sciences
Flinders University
Ph: +61 8 8201 3107

Dr Carly Moores (Project Manager)
Nutrition and Dietetics
Flinders University
Ph: +61 8 8201 5913

Dr Jacqueline Miller
South Australian Health and Medical Research Institute
Ph: +61 8 8128 4676

Dr Lucy Bell
Nutrition and Dietetics
Flinders University
Ph: +61 8 8204 5957

Dr Lucy Lewis
Physiotherapy
Flinders University
Ph: +61 8 7221 8261

Dr Ivanka Prichard
Health and Exercise Sciences
Flinders University
Ph: +61 8 8201 3713

Ms Aimee Macoustra
Nutrition and Dietetics
Flinders University
Ph: +61 8 8201 8845

Ms Naomi McKay
Nutrition and Dietetics
Flinders University
Ph: (c/o) +61 8 8201 5913

Description of the study:

This study is part of the project titled HOT – Health Online for Teens: Testing a new and experimental online program to improve teenage health. This project will investigate your use of the HOT online program, and whether you found it helpful. This project is supported by Flinders University, College of Nursing and Health Sciences

INSPIRING
ACHIEVEMENT

ABN 65 542 596 200, CRICOS No. 00114A

Purpose of the study:
This project aims to find out if teenagers use an online program which delivers health information about eating and activity. The program supports you to set and review eating and activity goals and provides nutrition and physical activity information and tips. This project also aims to ask you how you found the HOT program and whether it was useful for you.

What will I be asked to do?
You are invited to register for HOT and access program materials. You will be asked to participate in the HOT program over 14-weeks. You will review your current diet and level of activity, and set your own weekly goals to improve. There are weekly tips and quizzes, and resources such as recipes for you to use. Each week, you will review your progress against the goals you set. We will support you with strategies to overcome things that are making your changes hard. There will also be the opportunity to access support via an automated chat-bot in Facebook Messenger, which provides motivation and checks in on you each week. You will be issued with a mini iPad to use during this period and in appreciation of its safe return in good working order, you will receive a $50 gift card of your choice for Coles Myer or Apple iTunes/App Store.

For a period of 7 days both before and after the program, you will be asked to wear on your wrist an activity monitoring device (accelerometer). You will also be asked to complete two questionnaires, one before and one after the program which should take about 35 minutes each to finish.

You may also be invited to participate in an interview to discuss your thoughts about HOT and how you felt using the program. If you choose to participate, you will complete a one-on-one interview with a researcher and discuss your thoughts about HOT and how you felt using the program. The interview will be approximately 45 – 60 minutes long. The audio of interview will be recorded and later transcribed. You will have the opportunity to review the transcript and make changes to anything you've said.

Participation is entirely voluntary. You may use HOT without completing the survey at the end, and you can say no to participate in an interview when asked later.

ABN 65 542 596 200, CRICOS No. 00114A

What benefit will I gain from being involved in this study?
You will receive education and skills training to improve your diet and physical activity, as well as resources including goal setting tools.

If you choose to be interviewed, you may benefit by reflecting on your experience in HOT and your discussing any learnings from the HOT module with the researcher.

Will I be identifiable by being involved in this study?
After you complete the screening questionnaire, we will set up a HOT account/provide you a link to register. We recommend that your account be your first name and the first letter of your last name (e.g. John S or Jane T) so we can identify and contact you if there are any problems, but your identity (full surname) will be confidential and at the end of the program, your data will be linked, and linked with your parent's, by an ID number only (your name will be deleted from all records). You may not be anonymous to other members of the program if you share personal information or other details during the program.

Are there any risks or discomforts if I am involved?
The researcher anticipates few risks from your involvement in this study, however, given the nature of the project, some participants could experience emotional discomfort.

If participation in this study causes emotional discomfort (e.g., anxiety, distress, embarrassment), please call the Kids Helpline. Kids Helpline serves adolescents and young adults and is a free and confidential service which can be contacted any time by phone 1800 55 1800, email counsellor@kidshelpline.com.au, or web-chat https://kidshelpline.com.au/teens/get-help/webchat-counselling/.
If you have any concerns regarding anticipated or actual risks or discomforts, please raise them with the researcher.

There is a risk to your safety during the program and at all times when you are online if you provide personal details and information to others. We will protect your personal information from other participants and it is strongly recommended that you share personal details with care. Discussion forums will be monitored and we will make an effort to remove any revealing personal information which is shared. While all information provided will be treated with the strictest confidence, if serious indictable offences are witnessed or disclosed (e.g. child abuse), research staff as mandated reporters will need to report these

ABN 65 542 596 200, CRICOS No. 00114A

to the relevant authorities.

You will be loaned a new mini iPad for use during the program to ensure that you have access to a device on which to participate in the program. This is an expensive piece of equipment which will come with a protective case and it should be handled with care. You will not be financially responsible for replacing the device if it is damaged, but we do hope you take good care of it. If the device is returned in good working order, you will receive reimbursement for this care, and your time and participation in the study.

How do I agree to participate?
Participation is voluntary. If you are eligible to participate, you will be sent a registration link to the HOT program. Your enrolment survey and completion of registration is taken as your consent to participate. You are free to withdraw from the program at any time without effect or consequences.

Following the program, you may be asked to participate in an interview, which is voluntary. You will be given the opportunity to give consent to this interview by signing a consent form. You are free to say no to the interview or stop the interview at any time without effect or consequence.

How will I receive feedback?
On project completion outcomes of the project will be available to all participants via the Flinders University HOT website.

Thank you for taking the time to read this information sheet, and we hope that you will accept our invitation to be involved.

This research project has been approved by the Flinders University Social and Behavioural Research Ethics Committee (Project number 7896). For more information regarding ethical approval of the project the Executive Officer of the Committee can be contacted by telephone on 8201 3116, by fax on 8201 2035 or by email human.researchethics@flinders.edu.au

---

ABN 65 542 596 200, CRICOS No. 00114A

Flinders UNIVERSITY

PARENTAL CONSENT FORM FOR CHILD PARTICIPATION IN RESEARCH

Health Online for Teens – HOT Program

I …..

being over the age of 18 years hereby consent to my child ....

participating, as requested, in the interview for the research project on the Health Online for Teens – HOT Program.

1. I have read the information provided.
2. Details of procedures and any risks have been explained to my satisfaction.
3. I agree to audio recording of my child's information and participation.
4. I am aware that I should retain a copy of the Information Sheet and Consent Form for future reference.
5. I understand that:
   - My child may not directly benefit from taking part in this research.
   - My child is free to withdraw from the project at any time and is free to decline to answer particular questions.
   - While the information gained in this study will be published as explained, my child will not be identified, and individual information will remain confidential.
   - My child may ask that the recording be stopped at any time, and he/she may withdraw at any time from the session or the research without disadvantage.
   - My child will have the opportunity to review the transcript of the interview and make changes to their words.
   - There is a risk my child's safety during the program and at all times when they are online if they provide personal details and information to others.
   - While all information provided will be treated with the strictest confidence, if serious indictable offences are witnessed or disclosed (e.g., child abuse), research staff as mandated reporters will need to report these to the relevant authorities.

Participant's signature……………………………………Date…………………...

I certify that I have explained the study to the volunteer and consider that she/he understands what is involved and freely consents to participation.

Researcher's name………………………………………………………….................

Researcher's signature…………………………………..Date……………………

NB: Two signed copies should be obtained. The copy retained by the researcher may then be used for authorisation of Items 8 and 9, as appropriate.

ASSENT FORM FOR CHILD PARTICIPATION IN RESEARCH

Health Online for Teens – HOT Program

We are doing a research study about your experiences in our HOT program and what you thought about the program. A research study is a way to learn more about people.

If you decide that you want to be part of this study, you will be asked to be involved in an interview. The interview involves you sitting around and answer questions about your thoughts and feelings while doing HOT.

There are some things about this study you should know. The interview might take up to 60 minutes and your answers will be recorded. But this recording will not be shared with anyone other than the researchers.

Not everyone who takes part in this study will benefit. A benefit means that something good happens to you. We think you might benefit by talking and reflecting on your experience in HOT.

When we are finished with this study we will write a report about what was learned. This report will not include your name or that you were in the study.

You will have the opportunity to review the transcript of your interview which will have all of the words you've said in writing. You will be able to change some of what you've said if you like.

You do not have to be in this study if you do not want to be. If you decide to stop after we begin, that's okay too. Your parents know about the study too.

If you decide you want to be in this study, please sign your name.

I, ________________________________, want to be in this research study.

__________________________________ (Sign your name here)

__________________ (Date)

College of Nursing
& Health Sciences

Health Online for Teens
(HOT)

Nutrition and Dietetics
College of Nursing and
Health Sciences
Flinders University
GPO Box 2100
Adelaide SA 5001

HOT@flinders.edu.au

INFORMATION SHEET

for parents, HOT program

Title: Testing a new online program to improve teenage eating and lifestyle behaviours

Researchers:
Professor Michelle Miller
College of Nursing and Health Sciences
Flinders University
Ph: +61 8 8201 2421

Professor Anthony Maeder
Health Sciences
Flinders University
Ph: +61 8 8201 3107

Dr Carly Moores (Project Manager)
Nutrition and Dietetics
Flinders University
Ph: +61 8 8201 5913

Dr Jacqueline Miller
South Australian Health and Medical Research Institute
Ph: +61 8 8128 4676

Dr Lucy Bell
Nutrition and Dietetics
Flinders University
Ph: +61 8 8204 5957

Dr Lucy Lewis
Physiotherapy
Flinders University
Ph: +61 8 7221 8261

Dr Ivanka Prichard
Health and Exercise Sciences
Flinders University
Ph: +61 8 8201 3713

Ms Aimee Macoustra
Nutrition and Dietetics
Flinders University
Ph: +61 8 8201 8845

Ms Naomi McKay
Nutrition and Dietetics
Flinders University
Ph: (c/o) +61 8 8201 5913

Description of the study:
This study is part of the project titled HOT – Health Online for Teens: Testing a new and experimental online program to improve teenage health. This project will investigate your use of the HOT online program, and whether you and your child found it helpful. This project is supported by Flinders University, College of Nursing and Health Sciences

INSPIRING
ACHIEVEMENT

Purpose of the study:
This project aims to find out if teenagers and their parents use an online program which delivers health information about eating and activity. The program supports your child to set and review eating and activity goals and provides nutrition and physical activity information and tips. You will have access to parent program resources designed to help you support your child. This project also aims to ask you how you found the HOT program and whether it was useful for you and your child.

What will I be asked to do?
Your child is invited to register for HOT and access program materials. They will be asked to complete the HOT online program, which is 14-weeks long. You will have access to parent program materials during this time and will also be able to review the information and activities your child is completing each week.

We ask that you complete a short questionnaire before and after the program to help us get to know more about your family and child. Each of the two questionnaires will take approximately 30 minutes to complete.

At the end of the program, you may be invited to participate in an interview or focus group to discuss your thoughts about HOT and how you felt using the program. The interview/focus group will be approximately 45 – 60 minutes long, but focus groups may be longer and could take up to 90 minutes. The audio of interview/focus group will be recorded and later transcribed.

Participation is entirely voluntary. You can use HOT without participating in an interview or focus group when asked later.

What benefit will I gain from being involved in this study?
You will receive access to program resources to help support your child to improve their diet and physical activity.

Will I be identifiable by being involved in this study?
After you complete the screening questionnaire, we will set up a HOT account for you. We recommend that your account be your first name and the first letter of your last name (e.g. John S or Jane T) so we can identify and contact you if there are any problems, but your identity (full

ABN 65 542 596 200, CRICOS No. 00114A

surname) will be confidential and at the end of the program, your data will be linked, and linked with your child's, by an ID number only (your name will be deleted from all records). You may not be anonymous to other members of the program if you share personal information or other details during the program.

The interview/focus group will be recorded and your responses will be transcribed but names will be removed. You will not be identifiable in any results published from the study.

If you complete a focus group, it is possible that you may know other participants in the groups but confidentiality will be requested from all parties present before the start of the group discussion.

Are there any risks or discomforts if I am involved?
The researcher anticipates few risks from your involvement in this study, however, given the nature of the project, some participants could experience emotional discomfort.

If participation in this study causes emotional discomfort (e.g., anxiety, distress, embarrassment), please call Lifeline on 13 11 14 (http://www.lifeline.org.au/Get-Help/) or Beyond Blue on 1300 22 4636 (http://www.beyondblue.org.au). These are both free and anonymous counselling services. If you have any concerns regarding anticipated or actual risks or discomforts, please raise them with the researcher.

There is a risk to your safety during the program and at all times when you are online if you provide personal details and information to others. We will protect your personal information from other participants and it is strongly recommended that you share personal details with care. Discussion forums will be monitored and we will make an effort to remove any revealing personal information which is shared. While all information provided will be treated with the strictest confidence, if serious indictable offences are witnessed or disclosed (e.g. child abuse), research staff as mandated reporters will need to report these to the relevant authorities.

How do I agree to participate?
Participation is voluntary. You can agree to participate by logging in to your HOT account. You are free to withdraw from the program at any time without effect or consequences. You can decline the invitation to be involved in the interview/focus group and you are free to withdraw from the interview at any time without effect or consequences.

ABN 65 542 596 200, CRICOS No. 00114A

How will I receive feedback?
On project completion outcomes of the project will be available to all participants via the Flinders University HOT website.

Thank you for taking the time to read this information sheet, and we hope that you will accept our invitation to be involved.

This research project has been approved by the Flinders University Social and Behavioural Research Ethics Committee (Project number 7896) For more information regarding ethical approval of the project the Executive Officer of the Committee can be contacted by telephone on 8201 3116, by fax on 8201 2035 or by email human.researchethics@flinders.edu.au

---

ABN 65 542 596 200, CRICOS No. 00114A

Flinders UNIVERSITY

CONSENT FORM FOR PARTICIPATION IN RESEARCH

Health Online for Teens – HOT Program

I …..

being over the age of 18 years hereby consent to participate as requested in the interview for the research project on HOT – Health Online for Teens.

1. I have read the information provided.
2. Details of procedures and any risks have been explained to my satisfaction.
3. I agree to audio recording of my information and participation.
4. I am aware that I should retain a copy of the Information Sheet and Consent Form for future reference.
5. I understand that:
   - I may not directly benefit from taking part in this research.
   - I am free to withdraw from the project at any time and am free to decline to answer particular questions.
   - While the information gained in this study will be published as explained, I will not be identified, and individual information will remain confidential.
   - I may ask that the recording be stopped at any time, and that I may withdraw at any time from the session or the research without disadvantage.
   - I will be given the opportunity to review the transcript and make any changes to what I've said
   - There is a risk my safety during the program and at all times when I am online if I provide personal details and information to others
   - While all information provided will be treated with the strictest confidence, if serious indictable offences are witnessed or disclosed (e.g., child abuse), research staff as mandated reporters will need to report these to the relevant authorities
6. I agree/do not agree* to the transcript being made available to other researchers who are not members of this research team, but who are judged by the research team to be doing related research, on condition that my identity is not revealed.

** delete as appropriate*

7. I have had the opportunity to discuss taking part in this research with a family member or friend.

Participant's signature……………………………………Date…………………...

I certify that I have explained the study to the volunteer and consider that she/he understands what is involved and freely consents to participation.

Researcher's name…………………………………………………….................

Researcher's signature…………………………………..Date……………………

NB: Two signed copies should be obtained. The copy retained by the researcher may then be used for authorisation of Items 8 and 9, as appropriate.
