# Supplementary material for: A Digital Intervention for Australian Adolescents Above a Healthy Weight (Health Online for Teens): Protocol for an Implementation and User Experience Study
Source: JMIR Res Protoc. 2019 Oct 10;8(10):e13340. doi: 10.2196/13340 (PMC6913688; doi:10.2196/13340)
Supplement: Multimedia Appendix 3 [file resprot_v8i10e13340_app3.pdf]

Multimedia Appendix 2: Schedule of enrolment, interventions, and assessments (SPIRIT template)

	**STUDY PERIOD**																
	**Enrolment**	**Allocation**	**Post-allocation**														**Close-out**
**TIMEPOINT (weeks)**	$-t_1$	$-t_1$	1	2	3	4	5	6	7	8	9	10	11	12	13	14	$+t_{14}$
**ENROLMENT:**																	
Eligibility screen	×																
Informed consent	×																
Allocation		×															
**INTERVENTION:**																	
HOT		×	×	×	×	×	×	×	×	×	×	×	×	×	×	×	×
**ASSESSMENTS:**																	
Teens only:<br>- Self reported height and weight	×																×
Teens only:<br>- CDQ [41]<br>- Serves of core foods [42]<br>- Harter self-perception profile [47]<br>- 7-day accelerometry<br>- APARQ [45]<br>- ASAQ [46]		×															×
Parents only:<br>- CDQ [41]<br>- Harter self-perception profile [47]																	
Teens and parents:<br>-Engagement with HOT Moodle			×	×	×	×	×	×	×	×	×	×	×	×	×	×	
Teens only:<br>- Engagement with HOT-BOT			×	×	×	×	×	×	×	×	×	×	×	×	×	×	
Teens and parents:<br>- post-program satisfaction questionnaire<br>- Interviews																	×

Abbreviations: BMI, body mass index; CDQ, Children's Dietary Questionnaire; APARQ, Adolescent Physical Activity Recall Questionnaire; ASAQ, Adolescent Sedentary Assessment Questionnaire
